# Recently Emerged Swine Influenza A Virus (H2N3) Causes Severe Pneumonia in Cynomolgus Macaques

**DOI:** 10.1371/journal.pone.0039990

**Published:** 2012-07-11

**Authors:** Juergen A. Richt, Barry Rockx, Wenjun Ma, Friederike Feldmann, David Safronetz, Andrea Marzi, Darwyn Kobasa, James E. Strong, Lisa Kercher, Dan Long, Don Gardner, Douglas Brining, Heinz Feldmann

**Affiliations:** 1 Department of Diagnostic Medicine/Pathobiology, College of Veterinary Medicine, Kansas State University, Manhattan, Kansas, United States of America; 2 Laboratory of Virology, Public Health Agency of Canada, Winnipeg, Manitoba, Canada; 3 Office of Operations Management, Public Health Agency of Canada, Winnipeg, Manitoba, Canada; 4 National Microbiology Laboratory, Public Health Agency of Canada, Winnipeg, Manitoba, Canada; 5 Rocky Mountain Veterinary Branch, Division of Intramural Research, National Institute of Allergy and Infectious Diseases, National Institutes of Health, Hamilton, Montana, United States of America; 6 Department of Medical Microbiology, University of Manitoba, Winnipeg, Manitoba, Canada; 7 Department of Pediatrics and Child Health, University of Manitoba, Winnipeg, Manitoba, Canada; Centers for Disease Control and Prevention, United States of America

## Abstract

The triple reassortant H2N3 virus isolated from diseased pigs in the United States in 2006 is pathogenic for certain mammals without prior adaptation and transmits among swine and ferrets. Adaptation, in the H2 hemagglutinin derived from an avian virus, includes the ability to bind to the mammalian receptor, a significant prerequisite for infection of mammals, in particular humans, which poses a big concern for public health. Here we investigated the pathogenic potential of swine H2N3 in Cynomolgus macaques, a surrogate model for human influenza infection. In contrast to human H2N2 virus, which served as a control and largely caused mild pneumonia similar to seasonal influenza A viruses, the swine H2N3 virus was more pathogenic causing severe pneumonia in nonhuman primates. Both viruses replicated in the entire respiratory tract, but only swine H2N3 could be isolated from lung tissue on day 6 post infection. All animals cleared the infection whereas swine H2N3 infected macaques still presented with pathologic changes indicative of chronic pneumonia at day 14 post infection. Swine H2N3 virus was also detected to significantly higher titers in nasal and oral swabs indicating the potential for animal-to-animal transmission. Plasma levels of IL-6, IL-8, MCP-1 and IFNγ were significantly increased in swine H2N3 compared to human H2N2 infected animals supporting the previously published notion of increased IL-6 levels being a potential marker for severe influenza infections. In conclusion, the swine H2N3 virus represents a threat to humans with the potential for causing a larger outbreak in a non-immune or partially immune population. Furthermore, surveillance efforts in farmed pig populations need to become an integral part of any epidemic and pandemic influenza preparedness.

## Introduction

Influenza A virus infections in humans are typically associated with limited seasonal outbreaks of commonly circulating influenza virus strains. Occasionally however, new virus strains or subtypes appear that infect millions of individuals causing severe illness and high case fatality rates in humans [1]. So far four such influenza pandemics have been reported in 1918, 1957, 1968 and 2009 in the past 100 years [2].

Influenza A viruses can infect birds and a large variety of mammalian species including humans, horses, pigs, dogs, cats and sea mammals. Aquatic birds and shorebirds are considered natural reservoirs of influenza A viruses and 16 hemagglutinin (HA) and 9 neuraminidase (NA) subtypes have been isolated from these avian hosts [3]–[5]. In general, avian influenza viruses grow poorly in mammals including humans, cause little disease and are not easily transmitted between mammalian hosts [1]. Thus, only several subtypes of influenza A viruses have been established and maintained in mammalian species; for example, only three subtypes are known to have circulated in the human population (H1N1, H2N2 and H3N2) and only three subtypes of influenza A viruses (H1N1, H3N2 and H1N2) are consistently isolated from pigs worldwide. Pigs have been suggested to play an important role in transmission between birds and humans by acting as a “mixing vessel” for influenza viruses allowing for major genetic changes through reassortment of gene segments during co-infection [6], [7]. This capability may lie in the fact that viral receptors for both mammalian and avian viruses are present on porcine tracheal cells [8]. It is known that an avian-derived virus that infects and spreads among pigs can become adapted to growth in pigs and that swine-adapted viruses can readily be transmitted to humans as this might have happened with the 1918 pandemic [9], [10].

An H2N2 influenza virus, which emerged as a result of a reassortment event between circulating human H1N1 and avian H2N2 viruses, caused the Asian pandemic in 1957/58 with almost 2 million deaths worldwide [11]. This virus subtype disappeared from the human populations with the emergence of H3N2 virus that caused the Hong Kong pandemic in 1968 [12]. From 1968 to 2006, H2 subtype viruses were only detected in avian species with an Eurasian lineage genetically more similar to human H2 viruses than the American lineage [11], [13], [14]. However, some American lineage H2 viruses containing the HA from the Eurasian lineage as well as some Eurasian H2 viruses carrying PB2 and PA genes from the North American lineage have been isolated from shorebirds in North America [15] and from migratory ducks in Asia [16], respectively, indicating reassortment occurred between both H2 lineages.

In 2006, an H2N3 virus was isolated from pigs with respiratory disease in North America. This virus represents a reassortant between American avian viruses (H2, N3 and PA genes) and currently circulating North American swine influenza viruses [13]. It seems to be the first H2 virus that was isolated from a naturally infected mammal since 1968. The swine H2N3 caused typical interstitial pneumonia and acute necrotizing bronchiolitis in pigs and transmitted efficiently to sentinel animals. Mice inoculated with 10^4^ TCID_50_ or more of the H2N3 virus without prior adaptation showed labored breathing, rough fur, weight loss and lethargy; 75% of mice died when inoculated with 10^6^ TCID_50_. Although no obvious clinical symptoms were observed in ferrets, the H2N3 virus transmitted efficiently from infected ferrets to contact animals [13]. Therefore, this virus is already adapted to mammals and has acquired the ability to bind to the human/mammalian receptor, a highly significant prerequisite for the generation of an influenza virus that can infect and potentially transmit between humans. These observations raised concern for the potential of this newly emerged virus to cause a virulent outbreak in humans [13]. However, the pathogenicity and potential transmissibility of this new swine H2N3 virus in primates are unknown. Therefore, we infected Cynomolgus macaques (*Macaca fascicularis*), a surrogate model for human influenza infection as demonstrated before for infection with H5N1, 1918 and 2009 pandemic H1N1 influenza virus [17]–[22], with swine H2N3 to investigate its pathogenicity and transmissibility for nonhuman primates, using a 1957 pandemic H2N2 virus as a control.

## Results

### Disease progression in nonhuman primates

Twelve Cynomolgus macaques were infected using a previously established protocol [18] via four different routes (intratracheal, intranasal, oral and ocular) and a total dose of 7×10^6^ TCID_50_ of either human H2N2 or swine H2N3 virus. The challenge dose was confirmed by back-titration on MDCK cells (swine H2N3: 6.6×10^6^ TCID_50_/ml; human H2N2: 6.6×10^6^ TCID_50_/ml). Two animals from each group were euthanized at days 1, 6, and 14 post infection to specifically address the early disease stage, maximum pathology and recovery from infection, respectively, as well as allowing comparison to previous studies using the same disease model [18], [19], [21], [22]. All twelve animals rapidly reacted to infection with reduced food intake which lasted until 3–6 and 7–14 day post infection (dpi) for the human H2N2 and swine H2N3 infected group, respectively (Table S1). With the exception of the animals that were euthanized on 1 dpi, a smaller number of infected animals showed transitory signs of illness such as, reduced activity, elevated temperature and increased respiration rate (Table 1; Table S1). Disease signs were milder and of short duration for human H2N2-infected animals, whereas it was slightly more pronounced and lasted longer for swine H2N3-infected animals. Two animals in each group which were euthanized on 14 dpi had fully recovered from clinical signs of disease. All animals euthanized on 14 dpi and three of four animals euthanized on 6 dpi had influenza-specific antibodies and thus did seroconvert to infection. Animal #72-137 from the swine H2N3 group showed an equivocal antibody titer suggesting that if not euthanized at 6 dpi it would also have seroconverted (Table 1).

**Table 1:** Clinic, pathology and diagnosis.

Serotype	Animal No.	Sex (M/F)	Clinical signs^a^	Necropsy	Lower lobe (left/right)^b^	Middle lobe (left/right)^b^	Upper lobe (left/right)^b^	Diagnosis
H2N2	486	M	no	day 1	0/0	0/0	0/0	normal
	635	F	no	day 1	0/0	5/0	5/0	normal
	479	F	mild	day 6	0/0	0/0	0/0	normal
	134	M	moderate	day 6	0/0	0/50–75	0/50–75	pneumonia
	358^c^	M	mild	day 14	0/0	0/0	0/0	normal
	129^d^	M	mild	day 14	0/0	0/0	0/0	congestion
H2N3	805	M	no	day 1	0/0	0/0	0/0	normal
	637	F	no	day 1	0/0	0/0	0/0	normal
	937^e^	F	moderate	day 6	0/75	0/75	0/80	pneumonia
	72-137^e^	M	moderate	day 6	0/75	0/50	0/80	pneumonia
	745	F	moderate	day 14	0/75	0/90	0/65	pneumonia
	72-41^c^	M	moderate	day14	0/20	0/0	0/0	pneumonia

a clinical scoring was performed as published (21);

b gross pathology score in percentage of total lung tissue provided separately for the left and right lobe;

c animals with splenomegaly;

d all lung lobes were diffusely mildly reddened (distinct from other lesions);

e tracheo-bronchial lymph nodes moderately enlarged; F = female; M = male.

Physical examinations including blood pressure and pulse rate were normal throughout the study for all animals. Blood chemistry and hematology parameters were analyzed on every examination date. All animals showed transient leukocytosis, almost all neutrophils, on 1 dpi and fluctuations in glucose levels, both of which were interpreted as stress responses (data not shown). All other parameters showed no significant abnormal values and no differences within and between the two groups.

**Figure 1:**
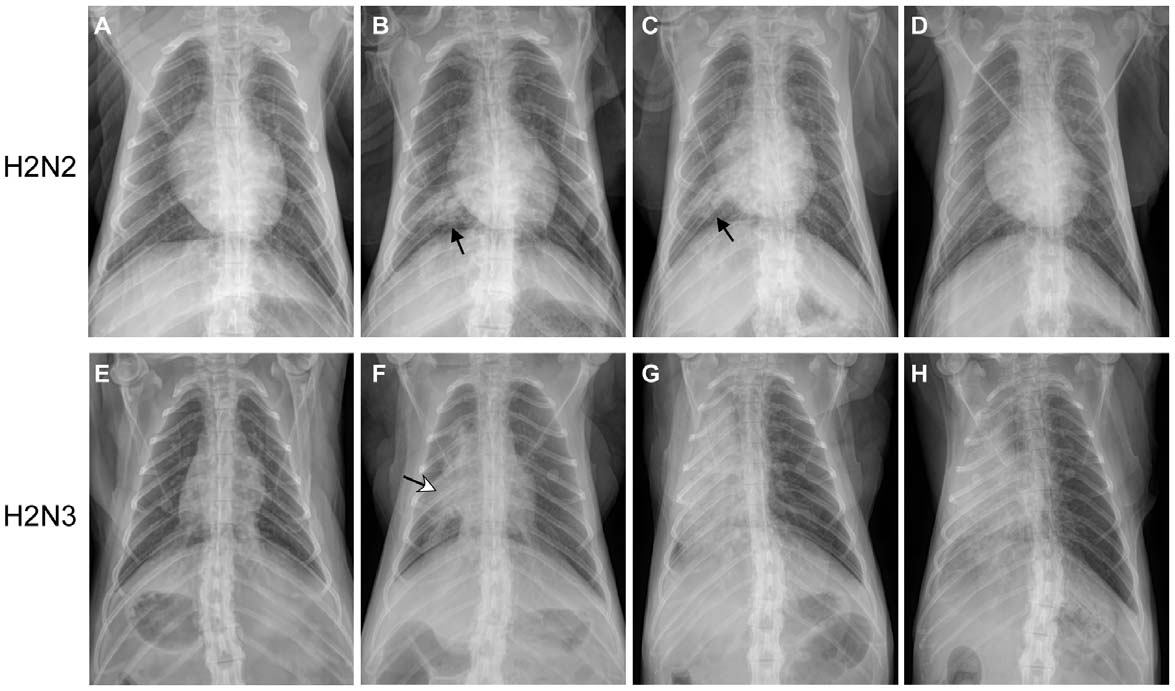
Radiographic imaging of disease progression. Time course ventrodorsal radiographs of representative macaques infected with either H2N2 or H2H3. Panels (A), (B), (C) and (D) represent time course radiographs from an individual animal at baseline, 3 dpi, 8 dpi, and 14 dpi infected with human H2N2 influenza virus. Focal interstitial radiographic changes (solid arrows) were noted on 3 dpi and 8 dpi but have resolved by 14 dpi. Panels (E), (F), (G) and (H) represent time course radiographs from an individual animal at baseline, 3 dpi, 8 dpi, and 14 dpi infected with swine H2N3 influenza virus. Focal interstitial changes with consolidation and partial effacement of the cardiac silhouette (open arrow) were noted. Radiographic changes are progressive on 8 dpi and 14 dpi with complete effacement of the cardiac silhouette and consolidation within the entire right hemi-thorax. Radiographic scoring was performed as previously published [21].

Disease progression was further monitored by radiographic imaging (X-ray) using a previously established scoring system [21]. For both human H2N2 and swine H2N3 infected macaques, the earliest signs of infiltration and interstitial markings were observed on 3 dpi (Figure 1). Human H2N2 infected macaques showed peak radiographic changes by 6 to 8 dpi and all had complete resolution by 14 dpi. Swine H2N3-infected macaques showed peak changes by 6 dpi and one animal had severe radiographic changes remaining until 14 dpi.

Overall, disease manifestation in animals infected with the human H2N2 virus was mild and in accordance with the limited changes seen on radiographic images. Interestingly, disease manifestation in animals infected with the swine H2N3 virus was only slightly more severe considering the quite dramatic infiltrations in several lobes of the right lung (Figure 1). The discrepancy might be explained by a predilection for infection of the right lung fields, which may be due to anatomic and experimental procedures used to infect an animal intratracheally, allowing the animals to still maintain reasonable respiratory function in unaffected areas of the left lung.

### Pathology in Cynomolgus macaques

**Figure 2:**
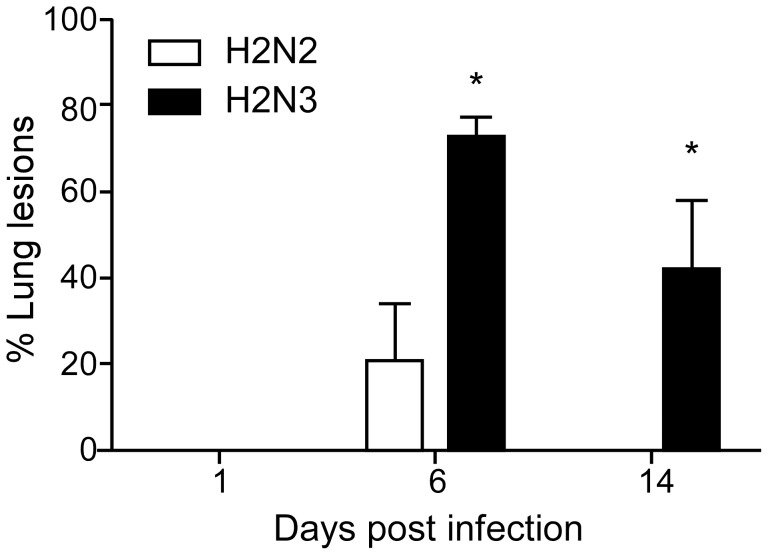
Gross pathologic lung lesions in H2N2 and H2N3 infected animals. Gross pathological lung lesions are expressed as the mean percentage of the upper, middle and lower right lung lobe surface covered by lesions (n = 2 per group). Error bars represent standard error of the mean. *significantly different from H2N2, P<0.01; 2-way analysis of variance (ANOVA).

Two animals from each group were euthanized at 1, 6, and 14 dpi to allow comparison to previous studies using the same disease model [18], [19], [21], [22]. Gross pathology revealed no abnormal tissues on 1 dpi for any animal. On 6 dpi one of the human H2N2 infected animals appeared normal in gross pathology, whereas the second H2N2 and both swine H2N3 infected animals showed evidence of pneumonia with extensive lesions in the right lung lobes. The day 14 necropsies revealed largely normal gross pathology for the H2N2 infected animals and evidence for chronic pneumonia in both H2N3 infected animals (Figure 2). The gross pathology supports clinical and radiographic observations of a milder disease in animals infected with human H2N2 virus and a more severe affliction of the right lungs in animals infected with swine H2N3 virus. With the exception of splenomegaly and enlarged tracheobronchial lymph nodes in 2 animals each (Table 1), all other organs appeared normal in gross pathology.

**Figure 3:**
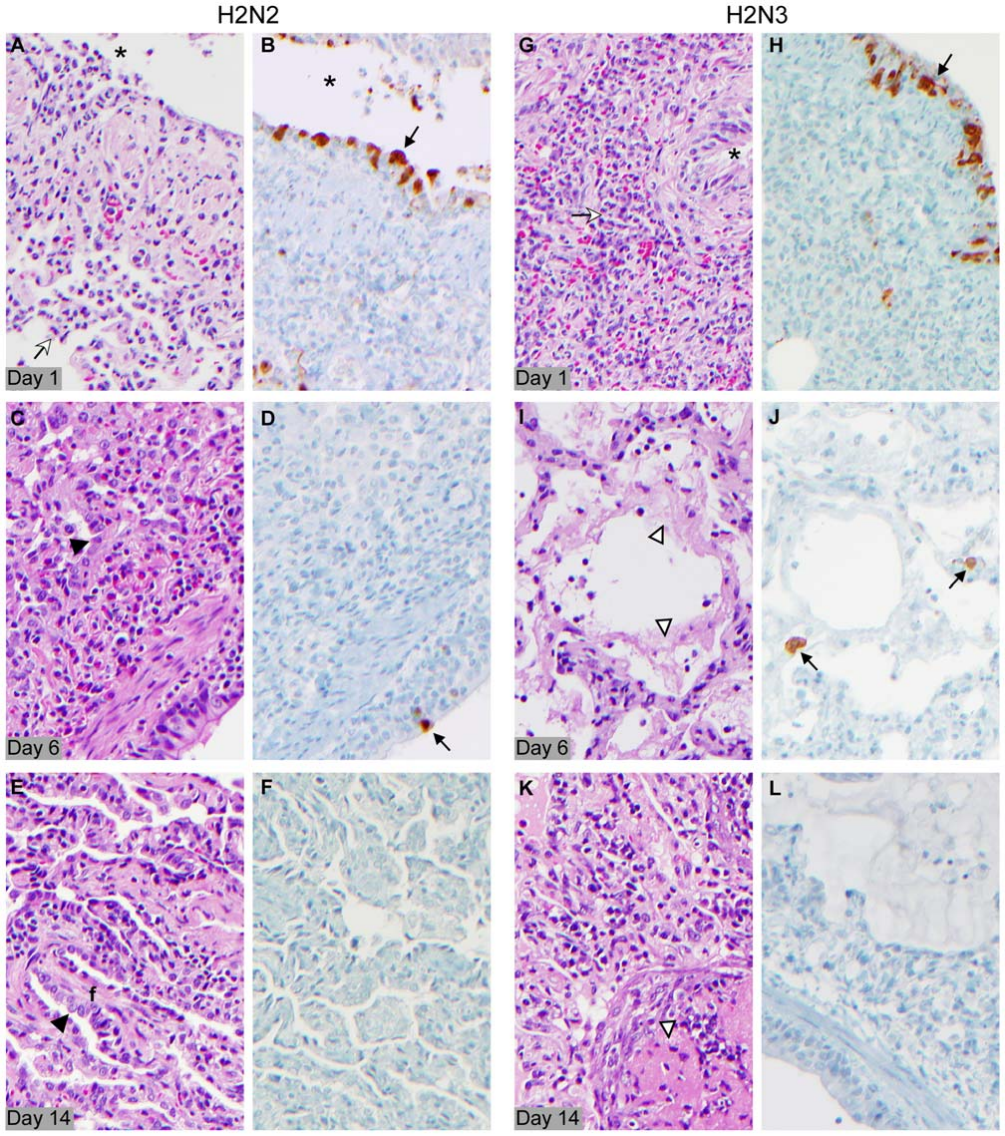
Histopathology of lung lesions. Histopathology was performed on samples derived from human H2N2 and swine H2N3 infected animals on 1, 6 and 14 dpi. The left hand columns (panels A, C, E, G, I, K) are H&E stained sections of lung representative of the three time points. The right hand columns are IHC stained lung sections from the same animal and lung lobe as the H&E stained sections. (A) Inflammatory cells (neutrophils and macrophages) and cell debris in a bronchial lumen (asterisk); similar cell population in adjacent alveoli (white arrow). (B) Antibody positive staining in cells of the bronchial lining epithelium with epithelial cell morphology (arrow). Asterisk indicates bronchial lumen. (C) Alveolar type II pneumocyte hyperplasia (black arrowhead) and fibrin in alveoli adjacent to a bronchus. (D) Rare antibody stained cell in bronchial lining epithelium (arrow). (E) Chronic pneumonia (fibroblasts, lymphocytes, plasma cells) with alveolar septal fibrosis (f) and type II pneumocyte hyperplasia (black arrowhead). (F) Absence of antibody staining. (G) Numerous neutrophils and macrophages within alveoli adjacent to a bronchus (white arrow). Asterisk indicates bronchial lumen. (H) Antibody positive stained cells in bronchial lining epithelium (arrow). (I) Fibrin hyaline membrane (open arrowhead); mixed inflammatory cells and thickened alveolar septae. (J) Rare antibody stained cells with macrophage or possible type II pneumocyte morphology (arrow). (K) Chronic pneumonia (fibroblasts, lymphocytes, plasma cells) and organized fibrin (open arrowhead) within alveoli. (L) Absence of antibody stained cells. Key: A, B = animal 635; C, D = animal 479; E, F = animal 358; G. H = animal 805; I, J = 937; K, L animal 745 (for animal numbers see also Table 1).

Based on previous experience with influenza A virus infection in this model [18], [22], histopathology was focused on lung tissues and analyzed by H&E stained materials. Day 1 post mortem lung lesions were similar for all animals in both groups and showed multifocal mild to moderate, acute to subacute bronchointerstitial pneumonia with multifocal necrosis of the bronchiolar lining epithelium (Figure 3A, G). Day 6 post mortem lung samples were also similar among all 4 animals in both groups. The most significant changes were characterized by moderate to severe, subacute to chronic pneumonia with extensive alveolar type II pneumocyte hyperplasia and multifocal alveolar fibrin and hyaline membrane formation (Figure 3C, I). In contrast to day 1 and day 6 specimens, we observed histologic differences on day 14 post mortem specimens between human H2N2 and swine H2N3 infected animals. H2N2 infected animals showed mild chronic pneumonia with fibrosis and type II pneumocyte hyperplasia as well as occasional squamous metaplasia of bronchiolar lining epithelium (Figure 3E). The swine H2N3 infected animals showed more severe chronic pneumonia with the same characteristics as mentioned above for the human H2N2 infected animals (Figure 3K).

Immunohistochemistry (IHC) staining for viral antigen was performed with a polyclonal anti-NP antibody (Figure 3). Viral antigen was detected on 1 dpi in alveolar pneumocytes, bronchial and bronchiolar epithelium and macrophages in animals infected with both viruses with no significant difference (Figure 3B, H). IHC staining on 6 dpi revealed staining of low numbers of bronchial and bronchiolar epithelial cells, alveolar type II pneumocytes and macrophages, with similar cells types and extent of IHC staining in both groups of animals (Figure 3D, J). Either no or very few antigen-positive cells were found in sections from day 14 specimens independent of the virus used for infection (Figure 3F, L).

Taken together, histopathology revealed no (1 dpi and 6 dpi) or only limited (14 dpi) differences in lung lesions between the two infected groups. However, the numbers and sizes of pulmonary lesions noted at necropsy were significantly increased for the animals infected with swine H2N3 virus with lesions affecting large areas of the right lung lobes which is consistent with the more severe clinical progression (Table 1, Table S1), the radiographic imaging (Figure 1) and the gross lung pathology (Figure 2) seen in these animals. Histologically, all animals had varying degrees of extent and severity of chronic pneumonia at 14 dpi when they had recovered from acute disease. Chronic pneumonia was more severe histologically in the lungs of one swine H2N3 infected animal (L-745) on 14 dpi. Histologic changes of the remaining three animals on 14 dpi were similar and comparable in extent and severity (Figure 3E, K).

### Virus replication in Cynomolgus macaques

**Figure 4:**
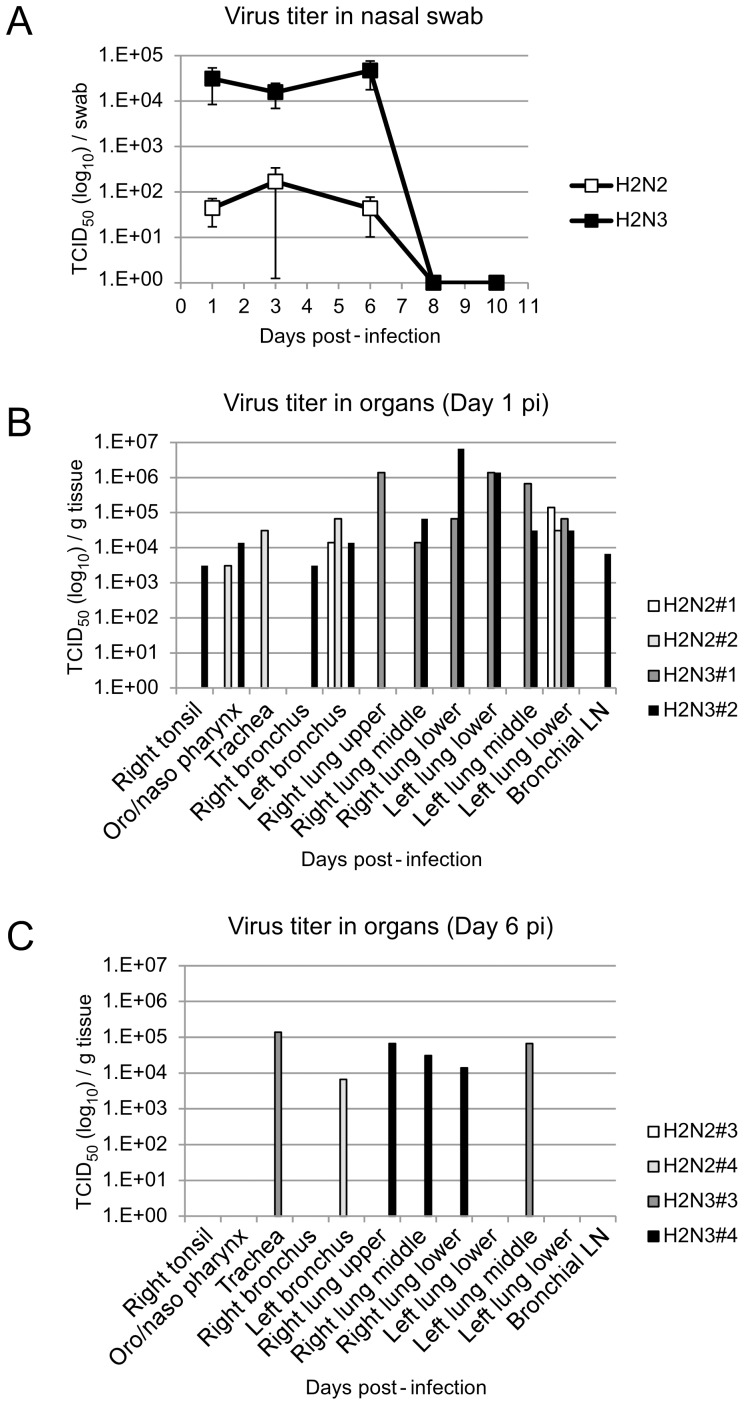
Virus replication in upper and lower respiratory tract. Virus titers were determined by TCID_50_ assay from nasal swabs and tissue specimens collected during examinations and necropsies, respectively. (A) This part shows titers of nasal swabs over time from human H2N2 and swine H2N3 infected animals. Error bars represent the standard errors of mean from 6 (1 dpi), 4 (3 dpi and 6 dpi) and 2 (8 dpi and 10 dpi) animals. A statistical significant difference in viral titers was observed between the groups by 2 way ANOVA (p = 0.0297). (B) and (C) These parts show titers (individual animals) from tissue specimens taken on 1 dpi and 6 dpi, respectively.

Assessment of virus replication was done in two steps, pre-screening with RT-PCR (data not shown) and final verification by virus titration from positive samples using a TCID_50_ assay in MDCK cells. Both the human H2N2 and swine H2N3 viruses were detected from the nasal cavity of infected animals. However, more animals infected with the swine H2N3 virus were positive for nasal swab samples and the virus titers were significantly higher (2–3 logs; 2 way ANOVA, p = 0.0297) when compared to the human H2N2 infected group (Figure 4A). Virus was only detected at low titers from the oral swabs collected from animals inoculated with the swine H2N3 virus on 3 dpi and 6 dpi; no virus was found in oral swabs collected from human H2N2 infected animals and rectal swabs collected from both groups of infection (data not shown). At 1 dpi the swine H2N3 virus was isolated from all lung lobes of both infected animals, whereas the human H2N2 virus was only isolated from the left lower lobe. Virus isolation from the upper respiratory tract tissue was inconsistent for both viruses at all time points (Figure 4B). Only the H2N3 virus could be isolated from lungs at 6 dpi (Figure 4C). Virus isolation was completely negative for all animals on 14 dpi.

Overall, this data indicates that swine H2N3 replicated more efficiently in the respiratory tract compared to human H2N2 consistent with the more progressive clinical presentation and pathology (Table 1, Table S1). Furthermore, swine H2N3 seems more likely to be shed from nasal mucosa but future experiments need to provide definite proof for a higher potential for transmission. The virus titer data confirms the histopathology findings (Figure 3) that all animals had cleared virus from their lungs by 14 dpi.

### Plasma cytokine response in Cynomolgus macaques

**Figure 5:**
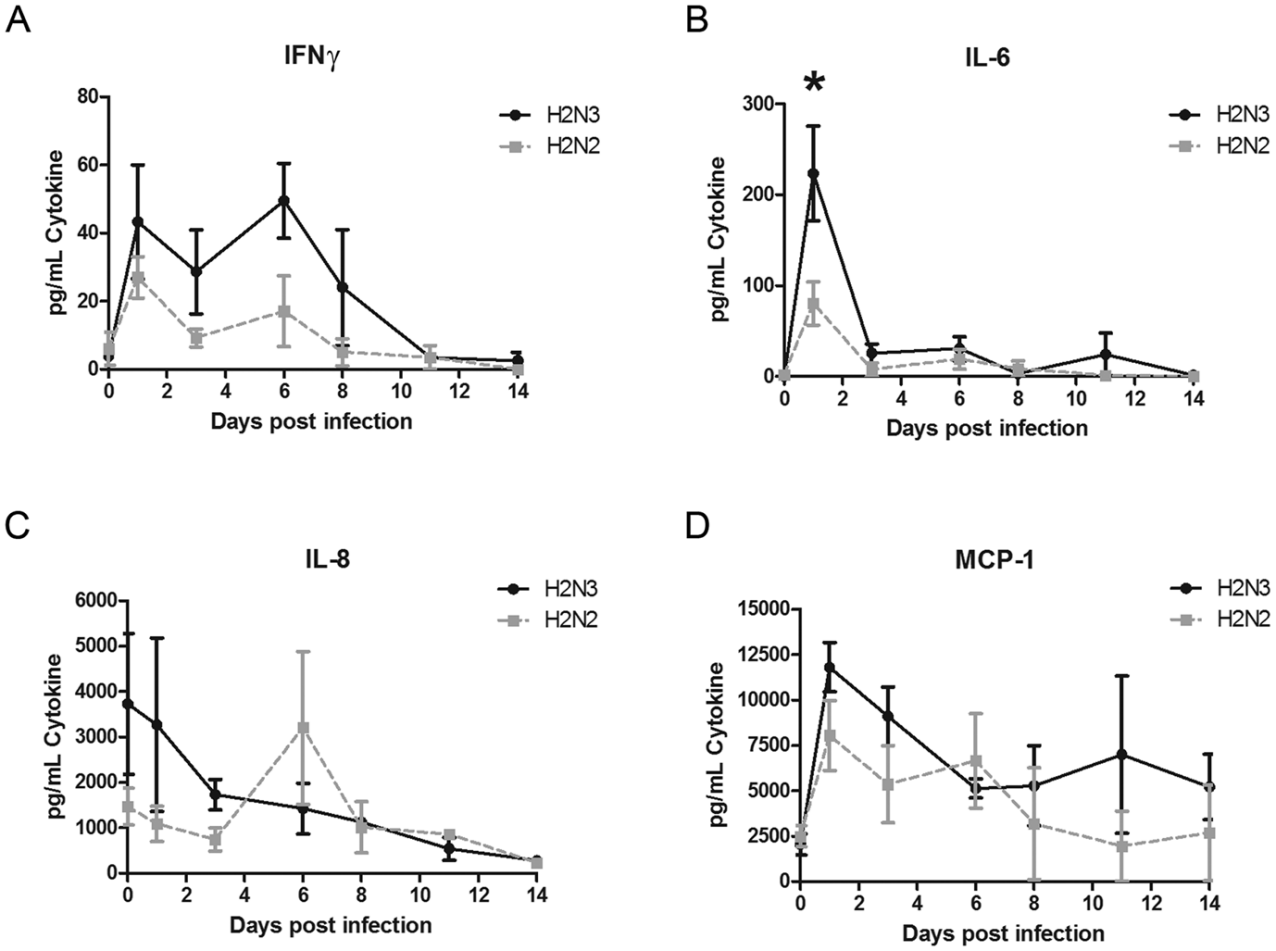
Plasma cytokine response in Cynomolgus macaques. Cytokine plasma levels were analyzed by a bioplex assay. (A) Interferon (IFN)γ, (B) Interleukin 6 (IL-6), (C) IL-8, and (D) Monocyte chemotactic protein 1 (MCP-1). Error bars represent the standard error of mean from 6 (1 dpi), 4 (3 dpi and 6 dpi) and 2 (8 dpi and 10 dpi) animals. *significant difference between H2N3 and H2N2 infected animals by t-test; p<0.05).

To study systemic host responses to infection we analyzed plasma cytokine levels according to the listing provided in ‘Materials and Methods’. We observed a trend of early increased IL-6, IL-8 and MCP-1 responses in animals infected with the swine H2N3 virus, which were less pronounced in animals infected with human H2N2 virus (Figure 5). Specifically, plasma levels of IL-6 were significantly higher in H2N3 infected animals compared to H2N2 on 1 dpi (T-test; p<0.05). The IFNγ response was increased in swine H2N3 infected animals over the first 8 days. All other tested cytokines did not differ in their plasma levels over time between the two groups on infection (data not shown). Overall, the cytokine levels in swine H2N3 infected animals were elevated compared to human H2N2 infections.

## Discussion

The H2N2 influenza A virus was responsible for the ‘Asian Flu’ pandemic of 1957/58 causing almost 2 million deaths worldwide including about 70,000 in the United States [23]. This virus is a reassortant between an avian (HA, NA and PB1 gene segments) and the previously circulating human seasonal H1N1 influenza virus (remaining 5 gene segments) [24], [25]. The pandemic H2N2 virus disappeared from the human population and was replaced by a human H3N2 virus which caused the Hong Kong pandemic in 1968 [12], indicating that people born after 1968 are unlikely to have preexisting immunity to an H2 subtype influenza virus. However, the H2 subtype still circulates in avian populations, especially shorebirds and migratory ducks, thus presenting a critical source for re-introduction of this serotype into mammalian populations including humans. The swine H2N3 virus isolated from US pigs with respiratory disease in 2006 is also a reassortant, in this case between an avian (HA, NA and PA gene segments) and endemically circulating swine influenza virus (remaining 5 gene segments), the latter of which is genetically similar to the most recent pandemic H1N1 virus [22], [26]. Interestingly, the swine H2N3 is pathogenic for pigs and mice without prior adaptation, an unusual feature for avian-derived influenza A viruses. In addition, this virus is transmissible amongst ferrets and pigs, indicting potential implications for public health [13].

Here we show that the swine H2N3 virus is able to infect Cynomolgus macaques with more efficient replication in the entire respiratory tract when compared to infection with the human pandemic H2N2 virus. The swine H2N3 virus caused pneumonia in the majority of infected animals (4/4; 2 animals were euthanized on day 1 post infection), whereas the human H2N2 virus resulted in pneumonia in only one infected animal (1/4; 2 animals were euthanized on 1 dpi), indicating that the H2N3 virus is more virulent and pathogenic for nonhuman primates than the human H2N2 virus and several seasonal influenza A viruses [18], [22]. Accepting the concept that the Cynomolgus macaque is a surrogate model for severe human influenza infection [18], [22], our results suggest that swine H2N3 might also be more virulent for humans. However, the lack of detectable exposure among pig workers during the swine epizootic in 2006 and no reported swine H2N3 infections in humans since 2006 do not support this hypothesis unless transmission from pig to human is highly inefficient [27]. Transmissibility may increase if swine H2N3 could establish itself in the human population, since this virus seems to have the potential for transmission among nonhuman primates based on positive nasal and oral swabs.

In comparison to other influenza A viruses studied in the Cynomolgus macaque model, H2N3 infection caused a moderate to severe pneumonia similar to what has been described for some of the early 2009 pandemic H1N1 strains [17], [22]. The disease is certainly more severe than infections with seasonal influenza viruses but less severe than described from infections with H5N1, 1918 or A/Mexico/InDRE4487/2009, one of the more virulent early pandemic H1N1 isolates [18], [19], [22]. Interestingly, clinical signs in swine H2N3 infected animals were considerably mild compared to relatively severe pathologic lesions found mainly in the right lung lobes (Table 1 and Figure 2). The left lungs seemed largely unaffected most likely allowing the animals to maintain a sufficient level of respiratory capacity and thus reducing clinical disease. This asymmetric distribution is likely explained by a combination of the technique used for intratracheal installation (animal lying flat on its back) and the anatomical features of the larger airways in the respiratory system of the Cynomolgus macaque favoring a preferential inoculation of the right lung.

Both the swine H2N3 and the human H2N2 viruses were detected in the nasal mucosa, trachea and bronchi of infected Cynomolgus macaques. However, the swine H2N3 virus replicated more efficiently in nasal mucosa and lung tissue of infected macaques supporting that it is adapted to mammalian species including primates as demonstrated before in challenge experiments of pigs, ferrets and mice [13]. The ability to productively infect mammalian species without prior adaptation occurs despite an HA protein that still contains an avian signature (glycine at HA position 228) [13]. However, the leucine at position 226 of the swine H2N3 HA protein might overcome this avian signature as this mutation has been associated with increased binding affinity to the mammalian-type receptor α2,6Gal-linked sialic acid [13]. The substitution D222G in the HA of 2009 pandemic H1N1 virus affects receptor binding and has been associated with cases of severe disease and high fatalities [28]. The H2N3 and H2N2 viruses used in this study both have 225G (H3 numbering system) which is conserved in human and avian H2N2 influenza viruses [13]. The role of the 225G in the HA of the H2 viruses needs to be investigated in future studies.

Severe influenza virus infection is associated with early inflammatory responses [18], [22], [29], [30]. In particular, increased IL-6 plasma levels have been discussed as an indicator for progression to more severe human influenza virus disease [31]–[33], it has been found after H2N3 infection and also during infections with more virulent pandemic H1N1 strains and the 1918 virus in macaques [18], [22]. IL-6 has been shown to play an essential role in protection against influenza virus infection by promoting neutrophil survival in the lung [34]. In addition, IL-6 has been identified as the main cause of fever in influenza infection [31]. Similarly, elevated plasma MCP-1 levels have been found in association with severe influenza infections in nonhuman primates and humans [18], [22], [33]. Specifically, MCP-1 may play a role in recruiting immune cells to the site of inflammation and clear alveolar epithelial damage. In contrast to infections with more severe pandemic H1N1 strains [22], the swine H2N3 virus caused early increased plasma IL-8 levels, a phenomenon that in general seems to be a signature of infections with less virulent influenza viruses [22], [35]. Elevated levels of IFNγ have been shown in H5N1-infected individuals as well as 1918 influenza-infected macaques [30], [36]. Overall, our results support the notion of IL-6, IFNγ and perhaps MCP-1 as potential indicators for more severe influenza disease.

In conclusion, swine H2N3 influenza A virus is virulent for several mammalian species without prior adaptation. This includes Cynomolgus macaques for which moderate disease and severe pathologic lesions were observed in this study. Pathogenicity of this virus in mammalian species might be associated with certain molecular features such as the leucine mutation at HA position 226 and/or 225G. In agreement with the increased transmissibility in mammalian models as described earlier [13], swine H2N3 also has a higher potential for shedding from the nasal mucosa of Cynomolgus macaques. Altogether, the emerged swine H2N3 virus represents a threat to humans with the potential of causing a larger outbreak (epidemic or pandemic) due to a partial lack of immunity in the human population. It also serves as a reminder for the emergence of human pathogenic influenza A viruses from the pig population as this was recently suggested with the H1N1 pandemic. Therefore, pig surveillance needs to be more intensified for proper animal and public health responses to influenza worldwide.

## Materials and Methods

### Viruses

The swine isolate A/Swine/Missouri/4296424/2006 (H2N3) [13] and the human isolate A/Singapore/1/57 (H2N2) (kindly provided by Dr. A. Garcia-Sastre, Department of Microbiology, Mount Sinai School of Medicine) were grown in MDCK cells, harvested at a cytopathogenic effect of 3+ (>70% rounded off and detached cells) for virus stock preparation, and titrated using a TCID_50_ assay on MDCK cells as described previously [18], [22]. All infectious work with influenza viruses was done under BSL3 conditions in the Integrated Research Facility (IRF) of the Rocky Mountain Laboratories (RML), NIAID, NIH.

### Animals and Ethical Statement

Healthy, adult female cynomolgus macaques (Macaca fascicularis) were handled in an ABSL3 containment laboratory at RML, DIR, NIH. Research was conducted in compliance with the Animal Welfare Act and other federal statutes and regulations relating to animals and experiments involving animals, and adhered principles stated in the Guide for the Care and Use of Laboratory Animals, National Research Council, 1996. The facility where this research was conducted (RML) is fully accredited by the Association for the Assessment and Accreditation of Laboratory Animal Care International and has an approved OLAW Assurance #A4149-01. Research was conducted under a protocol approved by the Institutional Animal Care and Use Committee (IACUC) at RML. All steps were taken to ameliorate the welfare and to avoid the suffering of the animals in accordance with the “Weatherall report for the use of non-human primates” recommendations. Animals were housed in adjoining individual primate cages allowing social interactions, under controlled conditions of humidity, temperature and light (12-hour light/12-hour dark cycles). Food and water were available ad libitum. Animals were monitored (pre- and post-infection) and fed commercial monkey chow, treats and fruit twice daily by trained personnel. Environmental enrichment consisted of commercial toys. All procedures were conducted by trained personnel under the supervision of veterinarians and all invasive clinical procedures were performed while animals were anesthetized. Early endpoint criteria, as specified by IACUC approved score parameters, were used to determine when animals should be humanely euthanized.

### Infection of Cynomolgus macaques

Twelve Cynomolgus macaques (*Macaca fascicularis*) (sex: 7 males and 5 females; age: ranging from 7–18 years; weight: ranging from 3.99–8.82 kg), previously tested seronegative for influenza viruses including H2 subtype by hemagglutination inhibition assay, were evenly divided in regards to age, sex and weight into two groups (6 animals/group). Animals were infected under anesthesia following an established protocol [18] through a combination of routes including intratracheal (4 ml), intranasal (0.5 ml per nostril), conjunctival (0.5 ml per eyelid) and oral (1 ml) with a suspension of either the H2N2 (*n* = 6) or H2N3 (*n* = 6) virus with a total infectious dose of 7×10^6^ TCID_50_ per animals. Animals were monitored daily for clinical signs (fever, posture, respiration, feces/urine, food intake, recumbence, attitude, and skin turgor) using an approved and previously published scoring sheet [21]. On −12, −4, 0, 1, 3, 6, 8, 11, and 14 dpi animals were examined under anesthesia at which point X-ray, pulse rate, blood pressure, temperature, and respiration rate were taken and each animal was bled for blood chemistry, hematology, virology, and cytokine profiles. In addition, oral, nasal and rectal swabs were taken on each examination date for virology. Two animals from each group were euthanized and necropsied on 1, 6 and 14 dpi with collection of clinical specimens from nasal mucosa, oral mucosa, conjunctiva, tonsils, cervical lymph nodes, trachea, bronchi, right and left lung (upper, middle, and caudal), hilar lymph nodes, heart, liver, spleen, pancreas, jejunum, transverse colon, kidney, adrenal gland, and mesenteric lymph nodes.

### Radiographic imaging and gross pathology scoring

X-ray images were scored blindly according to the following scheme: Grade 0 = normal examination; Grade 1 = mild interstitial pulmonary infiltrates; Grade 2 = moderate interstitial pulmonary infiltrates, may include partial cardiac border effacement, small areas of pulmonary consolidation; Grade 3 = pulmonary consolidation as primary lung pathology often seen as progression from Grade 2 [21]. For gross pathology the percentage of affected lung area was assessed in all lung lobes during necropsy by macroscopic examination [18], [22].

### Hematology and serum biochemistry

The total white blood cell (WBC) count, lymphocyte, platelet, reticulocyte and red blood cell counts, hemoglobin, hematocrit values, mean cell volume, mean corpuscular volume, and mean corpuscular hemoglobin concentrations were determined from EDTA blood with the HemaVet 950FS+ laser-based hematology analyzer (Drew Scientific, Waterbury, CT). Plasma biochemistry was analyzed from heparin blood using the blood chemistry analyzer iSTAT1 (Abbott Point of Care, Princeton, NJ). Urea nitrogen (BUN), glucose, chloride, sodium, potassium, hematocrit, hemoglobin, pH, PCO_2_, TCO_2_, base excess (BE_ecf_), and anion gap values were determined using the EC8+ Cartridge. Creatinine values were evaluated using Crea cartridges.

### Virus detection

Tissue samples were placed in RNAlater (Ambion) for subsequent RNA extraction (QIAGEN, RNAeasy kit). Real-time RT-PCR was performed as previously described [22] with the following primer/probe sequences: NP_forward_: gccataaggaccagaagtgg; NP_reverse_: tctgcattgtctccgaagaaata; NP_probe_: 6FAM-tttcgtccgagagctcgaagactcc-BBQ. RT-PCR was used to pre-screen swabs, blood, and organ samples for subsequent infectivity titration. For this, tissue homogenates (10% w/v) were prepared in Minimum Essential Medium (MEM) including bovine serum albumin (BSA). Debris was pelleted by centrifugation (2,000 g, 5 min.) and virus titers were determined in 10-fold dilutions of supernatant by standard TCID_50_ assay on MDCK cells [18], [22], in triplicate for each dilution. Virus titers were similarly determined in blood and swab suspensions.

### Plasma cytokine analysis

Concentrations of G-GSF, GM-CSF, IFNγ, IL-1β, IL-4, IL-5, IL-6, IL-8, IL-17, MCP-1 and MIP-1α in plasma of animals was determined on 0, 1, 3, 6, 8, 11, and 14 dpi using a Non-Human Primate Cytokine MILLIPLEX map kit (Millipore Corp., Billerca, MA) as described by the manufacturer. Samples were read using a Bio-Plex 200 system (Bio-Rad, Hercules, CA).

### Histopathology and Immunohistochemistry

Animal tissues were fixed in 10% phosphate-buffered formalin. Fixed tissues were processed by conventional methods, embedded in paraffin, cut into 5-µm-thick sections and stained with standard hematoxylin and eosin (H&E). Slides for immunohistochemistry (IHC) were stained using the Discovery XT and DAB Map Kit from Ventana Medical Systems (VMS), Tucson, AZ. For this a polyclonal rabbit anti-NP influenza antibody (kindly provided by Dr. Alan Goodman, Department of Microbiology, University of Washington) was diluted 1∶2,500 in Dilution Buffer (VMS) and incubated for 32 minutes. Following washing, a biotinylated secondary of goat anti-rabbit SS Link, (Biogenex, San Ramon, CA) was incubated for 32 minutes, followed by enzyme conjugate, and diaminobenzadine (DAB). Slides were counterstained with hematoxylin, dehydrated, cleared in xylene, and coverslipped.

### Serology

Serum samples collected prior to infection and on 1, 6 and 14 dpi were tested for influenza antibodies using the ‘Influenza A virus nucleoprotein (NP) antibody inhibition’ test kit (Virusys Corporation, Taneytown, MD). The test was performed according to the manufacturer's specifications and included both positive and negative controls (both provided in the kit).

## Supporting Information

Table S1 **Clinical data.**
(DOC)
