# Supplementary material for: Recently Emerged Swine Influenza A Virus (H2N3) Causes Severe Pneumonia in Cynomolgus Macaques
Source: PLoS One. 2012 Jul 11;7(7):e39990. doi: 10.1371/journal.pone.0039990 (PMC3394781; doi:10.1371/journal.pone.0039990)
Supplement: Table S1 — Clinical data. (DOC) [file pone.0039990.s001.doc]

# Supplementary Table 1: Clinical data

| **Animal ID (group)** | **Day 1 & 2** | **Day 3 - 6** | **Day 7 - 14** |
| --- | --- | --- | --- |
| 486 (H2N2 1 dpi) | food intake ↓ | n/a | n/a |
| 635 (H2N2 1 dpi) | food intake ↓ | n/a | n/a |
| 479 (H2N2 6 dpi) | food intake ↓↓ | food intake ↓↓ | n/a |
| 134 (H2N2 6 dpi) | food intake ↓ | food intake ↓ (until 4 dpi) | n/a |
| 358 (H2N2 14 dpi) | food intake ↓ | food intake ↓ | none |
| 129 (H2N2 14 dpi) | food intake ↓ | food intake ↓ | none |
| 805 (H2N3 1 dpi) | food intake ↓↓ | n/a | n/a |
| 637 (H2N3 1 dpi) | food intake ↓↓ | n/a | n/a |
| 937 (H2N3 6 dpi) | food intake ↓↓↓ respiration >40/min | food intake ↓↓↓  respiration >40/min (3 dpi) | n/a |
| 72-137 (H2N3 6 dpi) | food intake ↓↓ temperature of 39°C | food intake ↓↓ | n/a |
| 745 (H2N3 14 dpi) | food intake ↓↓↓ | food intake ↓↓↓ | food intake ↓↓↓ |
| 72-41 (H2N3 14 dpi) | food intake ↓↓↓ | food intake ↓↓↓ | food intake ↓↓↓ |

dpi = day(s) post infection;

none = no clinical signs;

n/a = not applicable;

↓ = food intake reduced by >50% of normal;

↓↓ = food intake reduced by >75% of normal;

↓↓↓ = no food intake at all

|  |  |  |  |
| --- | --- | --- | --- |
|  |  |  |  |
